# Assessing Age‐Associated Influences on Paramagnetic and Diamagnetic Susceptibility Maps in Postmortem Human Brains

**DOI:** 10.1002/nbm.70259

**Published:** 2026-03-25

**Authors:** José Henrique Monteiro de Azevedo, Maria Concepción Garcia Otaduy, Andre Avanzine, Roberta Diehl Rodriguez, Fábio Seiji Otsuka, Fernando Barbosa, Chunlei Liu, Carlos Ernesto Garrido Salmon

**Affiliations:** ^1^ InBrain, Department of Physics, FFCLRP University of São Paulo Ribeirão Preto Brazil; ^2^ Graduate Program Neurology, FMRP University of São Paulo Ribeirão Preto Brazil; ^3^ LIM 44, InRad, HC‐FMUSP University of São Paulo São Paulo Brazil; ^4^ Behavioral and Cognitive Neurology Group, Department of Neurology University of São Paulo São Paulo Brazil; ^5^ Analytical and System Toxicology Laboratory, Department of Clinical Analyses, Toxicology and Food Sciences, FCFRP University of São Paulo Ribeirão Preto Brazil; ^6^ Department of Electrical Engineering and Computer Sciences University of California, Berkeley Berkeley California USA; ^7^ Department of Medical Imaging, Hematology and Clinical Oncology, FMRP University of São Paulo Ribeirão Preto Brazil

**Keywords:** age, diamagnetic, paramagnetic, postmortem brain, QSM, susceptibility

## Abstract

Maps of paramagnetic and diamagnetic components of magnetic susceptibility can provide insights into the distribution of iron and myelin during brain aging. Postmortem validation is essential to ensure that these maps accurately reflect in vivo biological processes. In this study, we applied the APART‐QSM method for susceptibility separation to in situ (intracranial) postmortem MRI data from 47 subjects (ages 31–91) to investigate how age affects magnetic susceptibility components, comparing the results with previously reported in vivo associations. Linear regression was used to assess age‐related associations with susceptibility values in 17 deep gray matter (DGM) and white matter (WM) regions. Diamagnetic susceptibility showed a consistent age‐related decline in DGM basal ganglia regions, which appeared to result from a shared underlying factor across these areas. Based on the assumption that fractional anisotropy (FA) reflects myelin integrity, we also investigated the correlation between FA and diamagnetic susceptibility in WM regions. A negative correlation was found, suggesting a potential myelin contribution to the diamagnetic component. Consistent with in vivo analyses, the putamen's paramagnetic and total QSM susceptibility values demonstrated a strong age association in our postmortem condition, and it was the only region in which susceptibility values increased linearly with age. Finally, the analysis of ex vivo putamen tissue samples revealed a moderate association between paramagnetic susceptibility and iron concentration, supporting iron's biological contribution to MRI paramagnetic susceptibility maps of the putamen. The results enhance the biological interpretability of MRI data and promote cross‐validation between imaging and direct tissue analysis, with implications for both clinical and research applications related to aging and neurodegenerative diseases.

## Graphical abstract

We applied the APART‐QSM method to in situ postmortem MRI from 47 subjects (31–91 years) to assess age effects on paramagnetic and diamagnetic susceptibility. Diamagnetic susceptibility declined with age in basal ganglia, possibly reflecting shared biological factors. In white matter, it correlated with fractional anisotropy, suggesting myelin involvement. Putamen susceptibility increased with age, aligning with in vivo findings. Ex vivo analysis showed a moderate correlation between iron and paramagnetic susceptibility, supporting the biological relevance of the derived susceptibility maps.

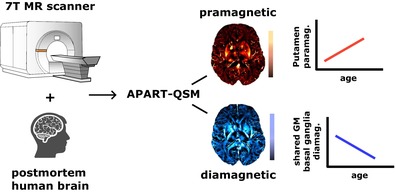

**Abbreviations**
DGM: deep gray matter
DTI: diffusion tensor imaging
FA: fractional anisotropy
GM: gray matter
ICP‐MS: inductively coupled plasma mass spectrometry
PMI: postmortem interval
QSM: quantitative susceptibility mapping
STAR‐QSM: streaking artifact reduction QSM
WM: white matter

## 1 Introduction

The aging process affects the human brain through a complex interaction of structural and molecular changes [1, 2]. Magnetic resonance imaging (MRI) techniques play a fundamental role in studying these effects [3]. Among them, quantitative susceptibility mapping (QSM) allows for the assessment of changes in composition and their associations with iron deposition throughout aging, by creating maps of magnetic susceptibility of the brain, which are related to iron accumulation [4].

Iron‐associated proteins, such as ferritin, the primary iron storage protein, are distributed throughout the brain, with higher iron accumulation in the deep gray matter (GM) regions, as found in chemical analyses of postmortem brain samples [5, 6] and by iron estimation using the QSM technique [4, 7]. Among these regions, the putamen stands out with a higher rate of susceptibility increase with progressive aging [8, 9].

While iron is the primary source of paramagnetic susceptibility in the brain, myelin is the primary source of diamagnetism [5, 10], which can be evaluated using diffusion tensor imaging (DTI). This technique can be used to estimate intravoxel fractional anisotropy (FA), a metric that quantifies the directional dependence of water diffusion, FA is particularly informative in white matter (WM) regions, where the organized structure of axonal fibers lead to anisotropic diffusion patterns. However, myelin is not the only source of diamagnetism. Some abnormal protein deposits, such as β‐amyloid plaques and neurofibrillary tangles, are also known to exhibit diamagnetic behavior [11]. Often, a colocalization of iron with β‐amyloid and p‐tau aggregations is found [11], indicating that excess iron can contribute to these abnormal protein aggregates. In addition, iron is important for myelin synthesis, being present in oligodendrocytes—the cells responsible for myelin production. Therefore, the colocalization of paramagnetic and diamagnetic substances in the brain may counteract each other in QSM, resulting in potential underestimation of true susceptibility values [12, 13, 14].

To overcome this limitation, recent methods have been proposed to separate these contributions. These methods rely on the assumption of a relationship between $R_{2}$ and susceptibility, as in the case of the χ‐separation method [12] and APART‐QSM [14]. APART‐QSM used the χ‐separation method as inspiration, treating the proportionality constant between $R_{2}^{\prime }$ and susceptibility as spatially varying to generate a more accurate separation. Some algorithms do not require the $R_{2}$ map, as in the case of DECOMPOSE [13], which fits multiecho data to a three‐pool model using only magnitude and phase data. Recently, APART‐QSM was used to identify developmental trajectories and the association of iron and myelin [15]. The authors detected patterns of myelination/demyelination in deep GM structures and iron influence in WM structures throughout aging in vivo.

While in vivo studies can provide valuable insights into understanding the effects of aging on the brain's paramagnetic and diamagnetic susceptibility, their findings must be validated to ensure that they accurately reflect biological processes, such as the true behavior of iron or myelin throughout aging. Postmortem brain MRI is the primary tool for validation, specifically, in situ MRI has the advantage of being an intermediate state between ex situ, which may have issues related to fixation, and in vivo, where performing many acquisitions may be too time‐consuming and uncomfortable for the person undergoing the scans. In addition, it allows direct correlation with histological results and providing a deeper understanding of the molecular changes observed in vivo [16].

However, reports validating paramagnetic and diamagnetic susceptibility maps are scarce, focusing on a limited number of brains with neurodegenerative diseases, and not targeting specific regions of the brain [17, 18]. Therefore, studies in specific regions, such as the putamen—which has stood out as the region with the highest susceptibility increase throughout aging—can be important to determine whether paramagnetic estimations align with total metal concentrations in the brain, and the observed age dependency of susceptibility is indeed reflected by the iron concentration changes.

In this cross‐sectional study, our goal is to apply the APART‐QSM method to MRI acquisitions of in situ postmortem brains from 47 subjects aged 31–91 years, to obtain paramagnetic and diamagnetic susceptibility maps and assess how these susceptibility components develop with age and their possible association with FA in several WM brain structures. Of these 47 subjects, 18 were used to obtain the total iron concentration in specific GM regions to determine whether the age dependency aligns with that observed in the susceptibility maps. As previous in vivo studies [8, 9], we hypothesize that the putamen will have the strongest association with age for both the paramagnetic component and iron concentration.

## 2 Methodology

### 2.1 MRI Acquisition and Image Processing

**FIGURE 1:**
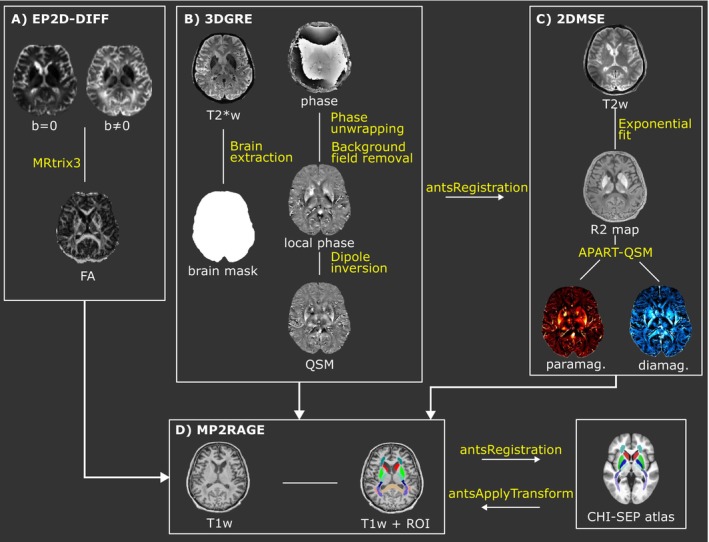
Pipeline for image processing of postmortem MRI brain data obtaining (A) FA maps, (B) QSM susceptibility maps, (C) paramagnetic and diamagnetic susceptibility maps, and (D) ROIs segmentation.

A total of 47 cases (age range at time of death: 31–91 years old, 19 females) were recruited from the City Death Verification Service of São Paulo after obtaining signed informed consent from the family and confirming no family‐reported signs of neuropsychiatric symptoms. MRI in situ images, that is, intracranium just before autopsy, of all these subjects were acquired just before the autopsy within a 24‐h postmortem interval (PMI). The research ethics committee of the Medicine School of the University of São Paulo (Approval Number 14407) approved this study. The main steps of the imaging processing pipeline from postmortem MRI data are shown in Figure 1.

#### 2.1.1 MRI Acquisition

The subjects were scanned using a Magnetom Siemens 7‐T scanner with a 32‐channel head coil. The following sequences were acquired: (1) 3D T1‐weighted magnetization prepared rapid gradient echo (MP2RAGE): repetition time (TR) 4000 ms, inversion time (TI1/TI2) = 2700/800 ms, echo time (TE) = 1.98 ms, flip angles = 5°/4°, voxel resolution = 0.8×0.8×0.8 mm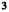, field of view (FOV) = 218×220 mm; (2) 3D multiecho gradient recalled echo (GRE): TR = 25 ms; TE = five equally spaced echoes between 5 and 21 ms, flip angle = 10°, voxel resolution = 0.5×0.5×1.0 mm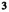, FOV = 203×224 mm; (3) 2D multiecho spin echo (MSE): TR = 15 ms, number of echoes = 10 equally spaced echoes between 15 and 150 ms, number of axial slices = 25, pixel resolution = 0.6×0.6 mm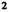, slice thickness = 3.3 mm, FOV = 179×229 mm; (4) diffusion‐weighted imaging (EP2D‐DIFF): diffusion gradients = 12; b values = 0, 1000, 3000, and 5000 s/mm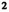; flip angle of refocusing pulse = 180°; pixel resolution = 2×2 mm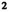; slice thickness = 2.2 mm; FOV = 216×220 mm; TE = 76 ms; TR = 6000 ms. Temperature was measured on the head during acquisition, with a maximum interindividual variation of 14°C.

#### 2.1.2 FA

Diffusion data of 44 subjects were processed with MRtrix Version 3.0.4 [19] and FSL Version 6.0.7.15 [20]. Out of the initial 47 subjects, three had no diffusion acquisition and were excluded from this analysis. The diffusion images were subjected to denoising and Gibbs ringing removal [21, 22, 23]. For correcting susceptibility‐induced distortions, and eddy currents FSL was used [24, 25]. To correct magnetic field uniformities, the ANTs N4 bias correction was applied in DWI images [26]. FA maps were obtained through fitting of diffusion tensor in each voxel, using MRtrix software. To obtain the mean FA value for each segmented structure, the b=0 image was registered to the 3D T1 space using a nonlinear transformation method implemented in the antsRegistration function of ANTs software [27]. This transformation file was applied to the FA map (Figure 1A).

#### 2.1.3 QSM Reconstruction

GRE phase images from all 32 channels were combined offline using the VRC method [28, 29]. The SEPIA toolbox (https://github.com/kschan0214/sepia) was used for QSM reconstruction. In summary, the raw phase of GRE was unwrapped using a Laplacian‐based phase unwrapping method. The tissue phase was obtained using the V‐SHARP method in the unwrapped phase [30]. The STAR‐QSM method was used for dipole inversion and susceptibility maps calculation [31]. As reference for the maps, cerebrospinal fluid mask, obtained by SEPIA, was used. The mask file was obtained using the magnitude image by applying the BET function in the SEPIA toolbox (Figure 1B).

#### 2.1.4 Paramagnetic and Diamagnetic Susceptibility Maps Reconstruction

A previous comparison between APART‐QSM [14] and DECOMPOSE [13] results from our data showed a good correlation for the paramagnetic susceptibility values but not for the diamagnetic components. This discrepancy might be due to the fact that our data were limited to five echoes, and the recommendation for the use of DECOMPOSE method is to use a larger number of echoes, because it relies on the fitting among the different TEs to extract the necessary T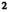 relaxation time, while the APART‐QSM method uses an extra acquisition for this. Therefore, considering that the diamagnetic component of APART‐QSM method already showed a good linear correlation with normalized myelin staining contents [14], and that it was previously applied to in vivo subjects [15], we decide to stick with APART‐QSM for our current data analysis.

The APART‐QSM method proposed by [14] was applied to GRE data to create the paramagnetic and diamagnetic susceptibility maps using MATLAB R2022b. This method employs a complex data model on the signal (Equation 1):

(1) $$\begin{array}{c} S(t)=M_{0}exp\{-\left(R_{2}+a\cdot \left|\chi_{\text{para}\;}\right|+a\cdot |\chi_{dia}\right|)t\}\cdot \\ exp\{i\left(\phi_{res}+\phi_{bg}(t)+2\pi \gamma B_{0}\left(D*\left(\chi_{\text{para}\;}+\chi_{\text{dia}\;}\right)\right)t\right)\} \end{array}$$

where a is the magnitude decay kernel, which is a proportionality constant between $R_{2}^{\prime }$ and absolute susceptibility sources with theoretical value of 754.8 Hz/ppm for 7 T, valid only on the static dephasing regime [13]; $\chi_{para}$ is the paramagnetic susceptibility; $\chi_{dia}$ is the diamagnetic susceptibility; $\phi_{res}$ is the time‐independent residual phase; B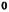 is the main magnetic field strength; $\phi_{bg}$ is the TE‐independent background phase; D is the magnetic dipole kernel; and * represents a spatial convolution.

To solve the complex model, an $R_{2}$map is necessary. This map was obtained using the ARLO function [32] implemented in MATLAB 2022a on the 2D MSE data. To mitigate intensity distortions that may influence the maps, the N4 Bias Field function from ANTs software [27] was used in 2D MSE images. Additionally, the model requires complete 3D GRE data of magnitude T2*‐w images, susceptibility maps, local phase, and the mask file. The local phase for each TE was obtained using the V‐SHARP method. A spatial linear transformation was applied to align the 3D GRE data with the 2D MSE space. This transformation involved two steps: first, manually aligning the brains using ITK‐SNAP to generate an initial transformation file and then applying antsRegistration software using this initial file to perform the final registration between the spaces. Finally, the APART‐QSM method was employed on the transformed data to obtain the paramagnetic and diamagnetic susceptibility maps. To obtain the mean susceptibility values in each segmented brain region, the susceptibility maps (QSM, paramagnetic, and diamagnetic) were transformed from native space to 3D‐T1w space. The transformation was achieved by applying the transformation files of the averaged echo 3D GRE magnitude image to the 3D T1‐weighted image for QSM maps and the first echo 2D MSE to 3D T1‐weighted image for paramagnetic and diamagnetic maps. ITK‐SNAP software was used to create an initial transformation in both cases, followed by registration using antsRegistration (Figure 1C).

#### 2.1.5 ROI Segmentation

**FIGURE 2:**
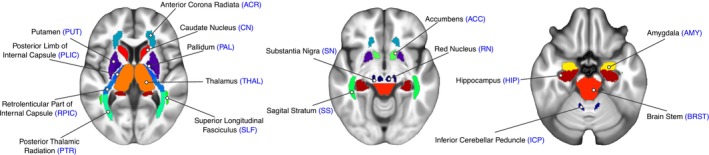
Representative ROIs used to analyze the age effects on magnetic susceptibility values.

MP2RAGE images underwent N4 Bias Field correction using the ANTs software [27]. Following correction, the FIRST‐FSL function was applied to automatically segment the brain regions: accumbens (ACC), caudate nucleus (CAU), pallidum (PAL), putamen (PUT), amygdala (AMY), hippocampus (HIP), and thalamus (THAL). Additionally, we have used a specific atlas in MNI space, that was constructed [33] to study myelin and iron distributions from susceptibility maps to obtain segmentation of specific WM and GM structures: inferior cerebellar peduncle (ICP), posterior limb of the internal capsule (PLIC), retrolenticular part of the internal capsule (RPIC), brain stem (BRST), anterior corona radiata (ACR), posterior thalamic radiation (PTR), sagittal stratum (SS), superior longitudinal fasciculus (SLF), substantia nigra (SN), and red nucleus (RN). The 3D T1 image was first nonlinearly transformed to the atlas space using ANTs, and then, an inverse transformation was applied to the segmentation in the atlas space to transform it back to the 3D T1 space (Figure 1D). All the segmented ROIs are illustrated in Figure 2.

For correlation of paramagnetic values and iron concentration, a manual segmentation of the putamen was performed using ITK‐SNAP on the coronally reconstructed MRI slices matching the histological slice from which the putamen was dissected, as described previously [34]. For this correlation, the tissue samples were separated in left and right hemisphere, so as the segmentation.

### 2.2 Iron Concentration Using Mass Spectroscopy

To obtain information on iron concentration in the putamen, tissue samples from the 16 available fixed brains (age range at time of death: 66.53 ± 15.91 years) among the 47 individuals initially considered were used. Tissue samples from the putamen were dissected by an experienced neurologist, lyophilized, and homogenized to create a powder‐like consistency. The homogenized tissue was then divided into three tubes, each containing 10 mg.

To measure iron concentration, the samples were solubilized following the methodology described by [35] for inductively coupled plasma mass spectrometry (ICP‐MS) analysis. The ICP‐MS readings were performed using a spectrometer equipped with a dynamic reaction cell (DRC‐ICP‐MS ELAN DRCII, PerkinElmer, SCIEX, Norwalk, CT, USA). Full specifications of the spectrometer, as well as the complete procedures for sample preparation and analysis, are detailed in [35]. Data validation was acquired by the analysis of Standard Reference Materials (SRM) 8414 and 1577b, from the National Institute of Standards and Technology (NIST). Nonsignificant statistical differences at 95% confidence level were observed between the found values and by applying a t test, demonstrating the accuracy of the proposed method.

For the comparison between paramagnetic susceptibility values and iron concentration, we included 18 subjects, where two subjects had known neurological conditions: one with an in vivo diagnosis of Parkinson's disease and another with moderate brain metastasis. We also evaluated the age dependence of PUT paramagnetic susceptibility and iron concentration, excluding the two cases with known neurological conditions.

### 2.3 Statistical Analysis

To identify the influence of age on the susceptibility values of postmortem brains, we performed a linear regression on the values obtained from QSM, paramagnetic, and diamagnetic maps. Linear regression was chosen as a suitable model for analysis considering the age range of our subjects and based on previous reports [6, 7]. Linear regression was also performed to analyze associations between iron concentration and paramagnetic susceptibility and also for iron concentration dependency with age.

To evaluate temperature effects in paramagnetic susceptibility, a general linear regression analysis considering age and temperature as covariates was also performed with 39 out of 47 available subjects.

Based on preliminary analysis of our data, the diamagnetic component appears to be the most affected by aging within basal ganglia. To investigate whether a common factor underlies the age‐related variations in diamagnetic susceptibility values across GM regions in basal ganglia, we conducted an exploratory factor analysis on these GM structures. The susceptibility data were first z‐score standardized across subjects for each ROI. The following basal ganglia ROIs were included: THAL, CAU, PUT, PAL, ACC, SN, and RN. Factor extraction was conducted in FactorAnalyzer Python module using principal axis factoring with a single‐factor solution and no rotation [36]. This procedure yielded one general factor score for GM for each subject, which we interpreted as the GM common factor.

To quantify region‐specific variance not explained by GM common factor, we computed the unique factors by subtracting the portion of each ROI's standardized value that was accounted for by the GM common factor (i.e., ROI z score minus the product of the factor score and the corresponding loading). Then, using the subtracted ROI values, we performed a new linear regression to identify if the previous diamagnetic associations with age persists.

The relationship between susceptibility and FA values of the postmortem brains was analyzed using the partial correlation coefficient to identify linear correlation, considering age as covariate.

Statistical significance was considered for p values < 0.05. The p values were corrected by multiple corrections using the Holm–Bonferroni method. All the statistical analysis was performed using Python 3.9.13.

## 3 Results

### 3.1 Representative Maps

**FIGURE 3:**
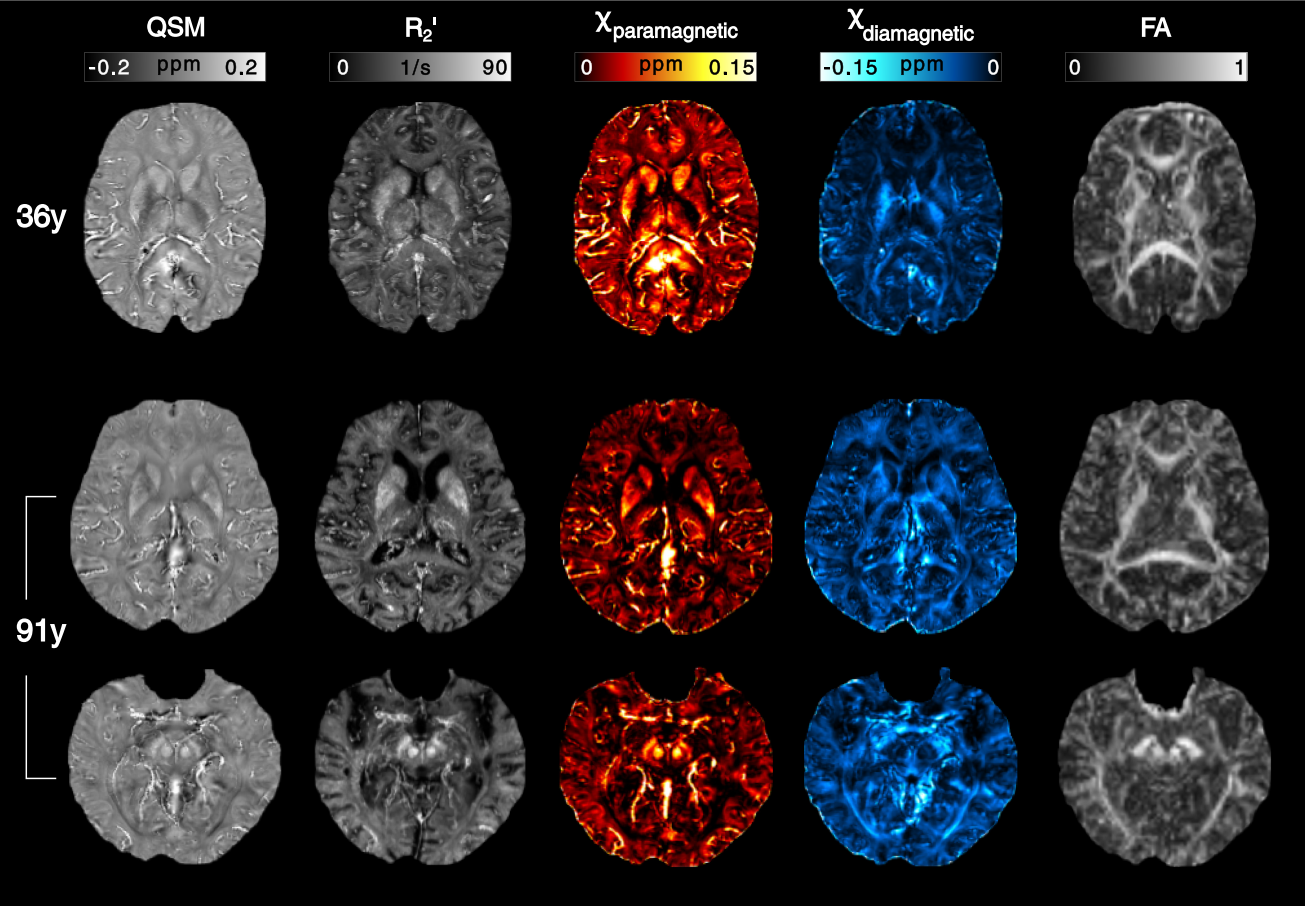
QSM, $R_{2}^{\prime }$, paramagnetic (APART‐QSM), diamagnetic (APART‐QSM), and FA maps of two representative postmortem brains at 36 (top) and 91 (two last rows for two different axial slices) years old.

The maps of QSM, $R_{2}^{\prime }$, paramagnetic, diamagnetic, and FA are shown from two representative subjects, 36 and 91 years old (Figure 3). Upon visual inspection, the paramagnetic maps show strong signals from vessels attributed to paramagnetic deoxyhemoglobin in the blood and strong signals in the regions of basal ganglia with the most iron concentration in the brain. In addition, it is possible to identify the regions associated with WM tracts by comparing the diamagnetic maps with the FA maps.

**FIGURE 4:**
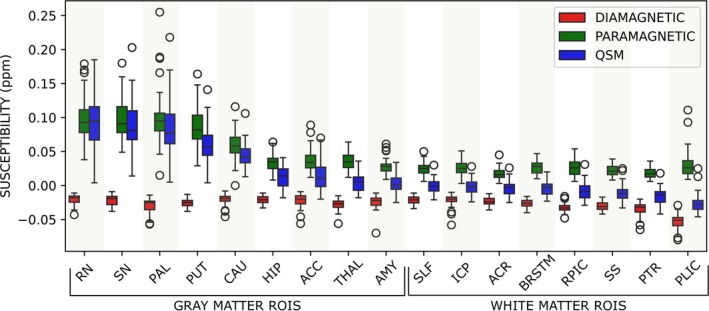
Mean from STAR‐QSM (QSM) and APART‐QSM (absolute value of diamagnetic and paramagnetic) for total (QSM) and partial susceptibilities in 17 brain regions. Vertical lines exclude outliers (max/min), and circles are outliers.

The diamagnetic component presented more stable values across the segmented regions than the paramagnetic ones, suggesting homogeneity in the biomolecular content associated to this component. This result can be observed in Figure 4 and in Table S4, which shows the mean, percentile, maximum, minimum values, and outliers of the susceptibility components for the brain regions studied among the subjects.

In addition, as expected by Curie law, temperature can affect paramagnetic susceptibility values, so we have performed a general linear regression analysis to observe any effects from temperature. This model was applied even when the expected variations in paramagnetic component of the susceptibility based on temperature variation were only 0.004 ppm for our interindividual variations, with a maximum of 14°C (19.83 ± 3.37). These expected variation values were estimated from a previous work in fixed tissue [13] and can be considered low compared to intervariations of the measured susceptibilities (Table S2). The magnitude decay kernel, a, can also be affected by temperature, as the APART‐QSM method spatially estimates the a, we also check the association of a with temperature, which, interestingly, showed a moderate association in PUT ($R^{2}=0.28$) and RN ($R^{2}=0.16$). On the other hand, temperature did not affect the FA values we found (Table S3).

### 3.2 Shared Basal Ganglia GM Factor Drives the Age Dependency of Diamagnetic Values

We have performed linear regression to identify the associations of the susceptibility values and aging. For the GM regions, the AMY, HIP, THAL and RN, no significative association with age of the susceptibility values for QSM, paramagnetic, and diamagnetic were found (Figures S8–S10). On the other hand, in PUT, we found a linear association with age for QSM, paramagnetic (Figure 6), and diamagnetic susceptibility values. Notably, beyond the PUT, we found a negative linear association with age for the diamagnetic susceptibility values in others GM basal ganglia regions: PAL, CAU, SN, and ACC, meaning an increase in diamagnetic species in older brains. This indicates that diamagnetic susceptibility is the most affected by aging.

Among the regions with significant linear associations, PAL exhibited the highest module rate of decrease in diamagnetic susceptibility values, while SN showed the lowest (Table S5). However, pairwise comparisons of the slopes did not yield statistically significant differences (p>0.05).

The identified basal ganglia GM common factor showed a moderate negative correlation with age ($R^{2}=0.46,p<0.001$), supporting the presence of a general trend of basal ganglia GM with age in diamagnetic susceptibility (Table 1). After controlling for this factor, the R mm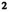 of these previous associations decreased and were no longer statistically significant.

**TABLE 1:** Slope of significant linear associations of diamagnetic susceptibility and aging process for accumbens (ACC), pallidum (PAL), substantia nigra (SN), putamen (PUT), and caudate nucleus (CAU). The $R_{before}^{2}$ was obtained before the consideration of a common factor for basal ganglia gray matter regions. After taking it into account, the $R^{2}$ for all structures decreases significantly ($R_{after}^{2}$).

Region	Slope (ppb/year)	$R_{before}^{2}$	$R_{after}^{2}$
Pallidum	−0.45	0.36	0.01
Accumbens	−0.31	0.22	0.01
Caudate	−0.29	0.30	0.02
Putamen	−0.24	0.32	0.00
Substantia Nigra	−0.23	0.18	0.00

*Note:* GM common factor: $R^{2}=0.46$, p<0.001.

### 3.3 FA Is Associated to Diamagnetic Susceptibility Values in WM Regions

**FIGURE 5:**
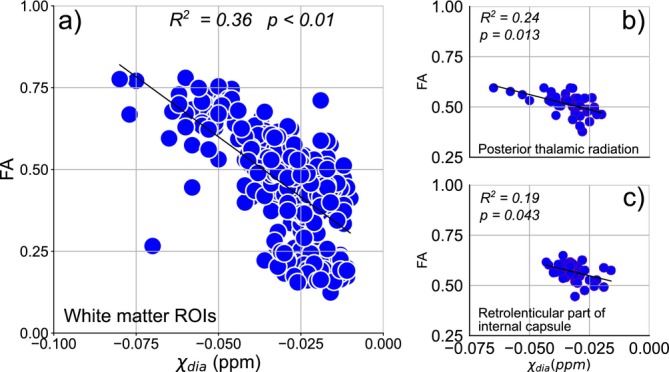
Correlation between diamagnetic components and FA values, using age as covariate, in (a) all white matter ROIs, (b) the posterior thalamic radiation, and (c) retrolenticular part of internal capsule.

To confirm influences of myelin on diamagnetic susceptibility values during aging, we analyzed the relationship between diamagnetic and FA values in all the WM regions studied. Because myelin content and integrity can also be indirectly inferred from FA values, we expected a negative association of FA with diamagnetic components. We found a negative linear association between FA and diamagnetic susceptibility, even when using age as a covariate ($R^{2}=0.36,p<0.001$) (Figure 5a). When performing the correlation analysis for individual WM ROIs, only two ROIs showed statistically significant association after correcting for age and multiple comparisons, the PTR ($R^{2}=0.24,p=0.013$) (Figure 5b) and RPIC ($R^{2}=0.19,p=0.043$) (Figure 5c). When checking this associations in CAU and ACC, we did not find any statistically significative associations (Figure S11).

### 3.4 Putamen Stands Out With Age‐Related Dependency in Magnetic Susceptibility

**FIGURE 6:**
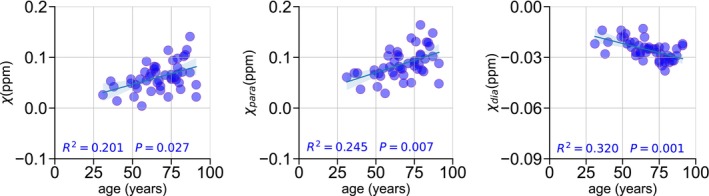
Linear regression of QSM (χ), paramagnetic ($\chi_{para}$), and diamagnetic ($\chi_{dia}$) susceptibility values and age for putamen.

The putamen was the only region where the QSM, and paramagnetic susceptibility values were linearly associated with age (Figure 6). In this region, isolating the paramagnetic from the diamagnetic component highlighted the age effect on the susceptibility value, which was 0.96 ppb/year, while the QSM showed a more modest increase with age, 0.84 ppb/year, due to the counteracting effect of the diamagnetic component.

**FIGURE 7:**
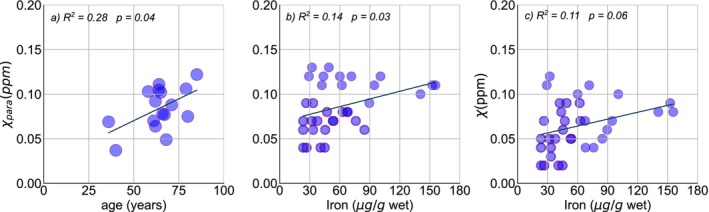
(a) Paramagnetic susceptibility dependency with age in putamen region of 16 subjects. (b) Paramagnetic susceptibility. (c) QSM and iron concentration correlation in putamen. Each point represents left or right hemispheres for 18 subjects, including one with Parkinson disease and one with moderate brain metastasis.

To evaluate the relationship of the PUT paramagnetic values with the total iron concentration in the tissue, as a function of age, we calculated the paramagnetic susceptibility for the PUT tissue dissected from the corresponding MRI images, separating left from right. We observed an increase in paramagnetic susceptibility as age increases for these subjects ($R^{2}=0.28,p=0.04$) (Figure 7a); however, iron concentration did not show a dependence with age.

We found a moderate association of the obtained paramagnetic values and the iron concentration ($R^{2}=0.14,p=0.03$) which improve after taking age as covariate ($R^{2}=0.22,p=0.004$) (Figure 7b). In addition, QSM values and total iron content show a lower association ($R^{2}=0.11,p=0.06$) (Figure 7c).

## 4 Discussion

This study used APART‐QSM method to analyze age‐related changes in magnetic susceptibility components in a cohort of 47 postmortem in situ brains aged 31–91 years. Consistent with previous reports [14, 15], we observed different spatial patterns in the susceptibility component age dependency in the GM regions studied. Therefore, the impact of age will be examined by categorizing structures that exhibit similar developmental trajectories in the subsequent sections.

### 4.1 Regions Without Linear Age‐Related Susceptibility Changes

Considering the magnetic susceptibility and age associations, no significant linear association was found in the AMY, THAL, BRST, HIP, RN, and WM regions. When analyzing our QSM susceptibility results, they are in agreement with in vivo findings for the AMY, THAL, and HIP. Howard et al. [37] analyzed the correlation between susceptibility values and age in these regions and did not find a significant correlation.

Previous QSM studies involving the THAL region and aging have shown conflicting results in literature, [38] observed a significant increase of QSM susceptibility values with in the THAL for in vivo subjects. Conversely, [39] reported a decrease in susceptibility values after peaking around 25 years, which is more consistent with Hallgren's postmortem histological data [6]. According to [4], this variability may be due to the THAL's high myelin concentration, where paramagnetism and diamagnetism affect conventional susceptibility values obtained using QSM. However, in our data, after separating the diamagnetic and paramagnetic susceptibility components, we did not find either a significant linear association with age in the THAL. This might indicate that, considering our analysis, the myelin content captured by diamagnetic susceptibility is not the primary driver of the conflicting QSM results previously reported. However, we cannot exclude the influences of our sample in this result, where unknown neurological conditions could be affecting the behavior of the magnetic susceptibility with age.

Moreover, we observed no significant linear association with age in the RN. Lao et al.'s study [15] for in vivo subjects indicated a slight increase in paramagnetic susceptibility with age after 30 years. We did not observe a comparable increase in our dataset, probably this effect is not sufficiently pronounced in our sample size. For QSM susceptibility values, [9] found a significant linear increase, while [15] found a slight increase with age in vivo. On the other hand, [7] reported stable behavior with age, similar to our results. Lao et al. [15] observed a more pronounced decrease in the diamagnetic component until about 35 years, followed by a plateau, which matches our findings.

Considering regions primarily composed of myelinated axons, [15] showed that QSM values in the PLIC decreased until around 30 years of age, then leveled off, similar to the diamagnetic component. The paramagnetic component, however, remained relatively stable with age. A similar trend was observed in the PTR, though the decrease in QSM and the diamagnetic component was slightly less pronounced [15]. In our study, we did not find any significant linear relationship between age and susceptibility components in the PLIC and PTR regions, consistent with the findings of [15], as our participants were aged 31 and above, where the QSM and diamagnetic component plateau was observed starting around 30 years of age. This result suggests that, for individuals aged 31 and above, both magnetic susceptibilities of these WM tracts present relatively stability.

### 4.2 Age‐Related Susceptibility Patterns in the Basal Ganglia

In the basal ganglia structures, the PAL, CAU, ACC, and SN shared a common finding: No significant linear association was found for QSM and paramagnetic components, indicating that in those regions, the iron concentration does not follow a simple linear trajectory.

The lack of significant association between QSM susceptibility values and age aligns with previous reports involving 135 [7] and 193 [8] in vivo subjects above 30 years. For paramagnetic susceptibility, visual comparison of data points from Lao et al.'s work [15] reveals similarities in the SN's paramagnetic susceptibility behavior above age 31. However, Lao et al.'s work indicates a slight increase in paramagnetic susceptibility in the caudate and pallidum above 31 years. This result differs from our observations. A possible source for this difference could be a negative effect size effect in our study, with the magnitude of the increase being so small that would be necessary a higher number of subjects to detect this effect.

However, for diamagnetic susceptibility, PAL, CAU, ACC, and SN show a significant negative linear association with age, similar to what was observed recently in vivo [40]. The age dependency in the PAL and CAU showed a similar visual trend to that observed in vivo by [15], indicating a slight decrease with age. In the SN, [15] found that diamagnetic susceptibility slightly decreases and then plateaus for ages above 30 years, a result that differs from our observations. This discrepancy could be due to differences in the selected ROI, as those authors only analyzed the posterior part of the SN.

Notably, the PAL, CAU, ACC, and SN are part of basal ganglia and have important connections to the brain function [41]. Our results show that the age‐related variations in diamagnetic susceptibility is affecting these regions in a similar way among them. When the effect of a common factor found for the diamagnetic component and age (see Section 3.2) in these ROIs was included as a covariate, the age association with diamagnetic susceptibility disappear, including the one found in PUT. This result suggests that the diamagnetic component exhibit a general age‐dependent trend in basal ganglia, possibly associated with the connections that they shared, which cannot be disregarded. As the APART‐QSM method uses the $R_{2}$ map to separate the susceptibility components, this result in basal ganglia regions could be biased by the dependency of $R_{2}$ on age, as shown previously [42], but the analysis of our data did not show any association between $R_{2}$ and age in these regions that could bias the trend we found for diamagnetic susceptibility.

This trend could be a global behavior of myelin in these structures, indicating a myelination process, which was described in vivo by [15], and/or a global effect of diamagnetic proteins in these regions, as it was previously raised the hypotheses that diamagnetic susceptibility increase in GM regions could be indeed be associated to accumulated plaques and tangles [43].

### 4.3 Association Between FA and Diamagnetic Susceptibility

The myelination hypothesis suggested by Lao et al.'s [15] work in GM of basal ganglia was made considering that the diamagnetic values are related to the myelin. Indeed, this association was previous observed using normalized myelin staining content in WM regions [14]. To further analyze this myelin association with diamagnetic component, FA can be used as an indirect measure of myelin content, as it shows a moderate association with myelin content estimated using myelin water fraction [44].

When performing a global analysis of all the ten WM regions in our study, FA values and diamagnetic susceptibility showed a significant negative correlation (Figure 5), even considering age as covariate: As diamagnetic susceptibility values become more negative (indicating increased diamagnetism), FA values increase. Despite not using age as covariate, a similar result was observed in a recent in vivo study [45]. This result supports the myelin influence for diamagnetic susceptibility values in WM structures. However, the relation for lower values seems dispersed—for FA, the lower values could indicate that factors other than myelin also play a role. In addition, we cannot exclude the influence of B0 orientation in relation to myelin fibers on the diamagnetic susceptibility values, which is not considered in the APART‐QSM model of susceptibility separation.

For individual ROI analysis, only the PTR and RPIC showed significant correlation after considering age as covariate. The lack of other individual ROIs correlation could be due to a limitation in our study concerning the 2DMSE acquisition, necessary to create the paramagnetic and diamagnetic maps. Our image acquisition was only in the central portion of the brain, around 24 slices. This acquisition restricted the ROI analysis, making it impossible to analyze the whole structures of the atlas used in some cases. Consequently, FA‐diamagnetic susceptibility associations in these structures remain undetermined in our study.

In addition, another limitation is that brain MRI was acquired with single channel transmission and no B1 maps were available to correct for possible B1 inhomogeneity impacts on $R_{2}$ calculation, but visual inspection of the images revealed that selected ROIs were included in the most B1 homogeneous part of the image. Also, a bias‐field correction using ANTs software was performed on 2DMSE images in order to mitigate this effect further.

On the other hand, analysis of DTI‐derived metrics on basal ganglia regions could be difficult. Basal ganglia regions have a higher rate of iron accumulation, which can cause an intense signal loss in diffusion‐weighted images, thereby influencing DTI‐derived metrics [9]. Indeed, in our preliminary analysis, the signal in diffusion‐weighted images of the PUT, SN, PAL, and RN showed signal intensities comparable with the background, corroborating the expected signal loss caused by higher iron accumulation and making the estimated FA values in these structures unreliable. In our analysis, the diamagnetic susceptibility values of PAL, CAU, SN, ACC, and PUT showed a decrease with age, also indicating a myelination process in aging. However, we could only check associations of FA and susceptibility values (including the diamagnetic) for CAU and ACC, which was found no significant (Figures S11–S13).

For GM of basal ganglia regions, the negative association of diamagnetic susceptibility and age, meaning an increasing in diamagnetic species as age increases, appears to be influenced not only by a myelination process. Diamagnetic proteins (e.g., β‐amyloid, tau proteins, and α‐synuclein) are known to increase with aging and affect susceptibility, increasing the diamagnetic influence [46]. As our analysis on CAU and ACC did not result in any significant association of diamagnetic and FA, our sample may have a significative influence of these proteins with diamagnetic behavior.

The accumulation of these proteins with age could be the biological basis of the age‐dependent factor observed across GM of basal ganglia [47]. Possibly the decrease in diamagnetic susceptibility values (i.e., increased diamagnetic effect) with age in the basal ganglia regions could be explained by this accumulation. Indicating that, when analyzing magnetic susceptibility findings in these regions, not only the effect of iron contained proteins should be considered but also of others' proteins with diamagnetic behavior. However, we should not exclude the crosstalk effect from iron accumulation affecting the diamagnetic changes in basal ganglia regions. The complex biophysics in these regions, with the possibility of having multiple signal sources, for example, iron, myelin, and diamagnetic proteins, that are not all assumed in the model, can influence the interpretation of the finding in these regions.

### 4.4 Magnetic Susceptibility and Iron Content of Putamen Across Age

The PUT, the largest region of the basal ganglia nuclei, showed unique results in our analysis. As we expected, based on previous in vivo studies [8, 9, 15], it was the only region where we observed a positive linear association for QSM and paramagnetic values with age. The QSM susceptibility behavior in the age range of 25–78 years, as obtained by [9] exhibited a similar trend to our results. They found the highest rate of iron accumulation with age in the PUT (slope of linear regression = 0.73 ppb/year), comparable with our findings for postmortem brains (Table S4). Lao et al. [15] also reported similar findings; although they used an exponential growth model for QSM and paramagnetic susceptibility values, they observed a continuous increase at an approximately constant rate with age, similar to [8]. The results for the diamagnetic component reported by [15], using in vivo data and an exponential growth model, indicated a gradual decrease after about 20 years, reaching a plateau with increasing age. In our study, the diamagnetic component showed a significant but slight linear decrease. However, this behavior of the diamagnetic component was associated with a common factor present in GM regions within the basal ganglia.

Comparing our QSM and paramagnetic susceptibility values after separating susceptibility components, we found that, despite the difference not being statistically significant, the paramagnetic susceptibility values showed a higher linear increase with age. This may indicate that QSM values are substantially affected by an age‐independent diamagnetic effect, which ends up underestimating the age dependency driven by the paramagnetic component.

The increase of paramagnetic susceptibility with age in the PUT could indicate a higher rate of iron in this region. However, our ICP‐MS results of total iron concentration in the PUT of 16 subjects did not show an association of iron with age, while it was possible to find a significant positive correlation with age of the PUT paramagnetic susceptibility in the same subjects. A previous study with 42 postmortem brains, with an age interval of 53–101 years old, has found a linear association of iron concentration, obtained using ICP‐MS, with age in the putamen [48], when comparing our results for the putamen, they are not in agreement. Differences in methodology, tissue processing, lower number of subjects, and anatomical precision may all contribute to the observed differences between previous results and our findings. In addition, while we consider subjects with no documented neuropsychological conditions, we cannot assure that brain alterations that were not yet affecting significantly the in vivo subject could not be significative in this analysis. This divergence within the same subset of subjects suggests that while paramagnetic susceptibility in the PUT is influenced by iron, its age‐related increase might reflect factors beyond just total iron concentration, such as changes in iron's chemical form or the contribution of other paramagnetic species not accounted for by total iron measurement.

We found a positive but only moderate correlation between paramagnetic susceptibility and iron concentration, indicating that the iron concentration is influencing the paramagnetic susceptibility component, but this can be influenced by others factors, such as the fixation time, that in our cases were about 2 and 3 years, and previous work [5] was about 5 weeks. Nonetheless, the correlation strength we observed (Figure 7) is comparable with results from another study in patients with epilepsy, where laser ablation‐ICP‐MS was used to quantify iron from brain tissue samples of the epileptogenic zone [17].

## 5 Conclusions

This study examined how aging affects paramagnetic and diamagnetic susceptibility in in situ postmortem human brains using the APART‐QSM method. A consistent age‐related trajectory of diamagnetic susceptibility across basal ganglia GM regions suggests a common underlying factor, likely related to diamagnetic proteins rather than solely myelination. The putamen showed distinct age‐dependent changes in both QSM and paramagnetic susceptibility, reflecting known in vivo patterns of paramagnetic accumulation. Although total iron concentration in the putamen did not correlate with age in our sample, a moderate association with paramagnetic susceptibility was observed, suggesting that iron contributes to its paramagnetic signal. Overall, postmortem susceptibility trends closely mirror in vivo findings, supporting the use of postmortem magnetic susceptibility maps to study neurodegenerative processes and enhance magnetic susceptibility source separation methods.

## Author Contributions

**José Henrique Monteiro de Azevedo:** formal analysis; methodology; investigation; data curation; visualization; writing – original draft. **Maria Concepción Garcia Otaduy:** conceptualization; resources; funding acquisition; project administration; writing – review and editing. **Andre Avanzine:** software; data curation. **Roberta Diehl Rodriguez:** investigation; writing – review and editing. **Fábio Seiji Otsuka:** investigation; software; writing – review and editing. **Fernando Barbosa Jr.:** investigation, writing – review and editing. **Chunlei Liu:** investigation, writing – review and editing. **Carlos Ernesto Garrido Salmon:** conceptualization; methodology; supervision; project administration; funding acquisition; writing – review and editing.

## Funding

The research reported in this publication was supported in part by the National Institute on Aging of the National Institutes of Health under Award Number R01AG070826, Coordenação de Aperfeiçoamento de Pessoal de Nível Superior—Brasil (CAPES)—Finance Code 001 (J.H.M.A. scholarship), São Paulo Research Foundation (FAPESP, project: 2023/04823‐3, A.A. scholarship; 2021/05059‐0, F.B.Jr. Multi‐User Equipment Program grant), and Brazilian National Council for Scientific and Technological Development (CNPq, project: 310618/2021‐5, C.E.G.S. fellowship).

## Conflicts of Interest

The authors declare no conflicts of interest.

## Supporting information



supplementary.pdf

## Data Availability

The data that support the findings of this study are available from the corresponding author upon reasonable request.
