# Supplementary material for: Assessing Age‐Associated Influences on Paramagnetic and Diamagnetic Susceptibility Maps in Postmortem Human Brains
Source: NMR Biomed. 2026 Mar 25;39(5):e70259. doi: 10.1002/nbm.70259 (PMC13018726; doi:10.1002/nbm.70259)
Supplement: Supplementary file 1 — supplementary.pdf [file NBM-39-e70259-s001.pdf]

Supplementary Information

Table 2: General linear model results using age and inverse of temperature as co-variables to try identify influences on the paramagnetic susceptibility values obtained using APART-QSM method. Significant association is indicated in bold

Variable	Coef._{age}	S.E._{age}	t-value_{age}	$p_{corr.age}$	$\text{Coef.}_{1/T}$	$\text{S.E.}_{1/T}$	$\text{t-value}_{1/T}$	$p_{corr.1/T}$	R^2
THAL	-8.42e-05	1.61e-04	-5.23e-01	1.00e+00	-9.79e+01	6.21e+01	-1.58e+00	1.00e+00	6.92e-02
CAU	3.03e-04	2.37e-04	1.28e+00	1.00e+00	-1.27e+01	9.13e+01	-1.39e-01	1.00e+00	4.70e-02
**PUT**	**1.04e-03**	**2.94e-04**	**3.53e+00**	**1.99e-02**	-7.56e+01	1.13e+02	-6.66e-01	1.00e+00	2.81e-01
PAL	-7.73e-06	3.44e-04	-2.25e-02	1.00e+00	4.02e+00	1.33e+02	3.03e-02	1.00e+00	4.58e-05
BRST	-2.27e-05	1.07e-04	-2.11e-01	1.00e+00	-8.37e+01	4.15e+01	-2.02e+00	7.69e-01	1.04e-01
HIP	1.28e-04	1.64e-04	7.79e-01	1.00e+00	-1.32e+02	6.32e+01	-2.09e+00	7.04e-01	1.34e-01
AMY	1.91e-04	1.17e-04	1.63e+00	1.00e+00	-1.21e+02	4.50e+01	-2.69e+00	1.87e-01	2.40e-01
ACC	1.19e-04	1.93e-04	6.19e-01	1.00e+00	-5.63e+01	7.44e+01	-7.56e-01	1.00e+00	2.99e-02
SN	-7.44e-04	2.83e-04	-2.63e+00	2.00e-01	-5.23e+01	1.09e+02	-4.80e-01	1.00e+00	1.66e-01
RN	1.52e-04	3.54e-04	4.30e-01	1.00e+00	-2.47e+02	1.36e+02	-1.81e+00	1.00e+00	9.56e-02
ICP	9.07e-05	1.16e-04	7.81e-01	1.00e+00	-6.68e+01	4.48e+01	-1.49e+00	1.00e+00	8.24e-02
PLIC	2.18e-04	2.05e-04	1.06e+00	1.00e+00	-8.03e+01	7.90e+01	-1.02e+00	1.00e+00	6.53e-02
RPIC	2.04e-05	1.30e-04	1.57e-01	1.00e+00	-6.71e+01	5.00e+01	-1.34e+00	1.00e+00	5.15e-02
ACR	1.38e-05	1.02e-04	1.35e-01	1.00e+00	-4.77e+01	3.93e+01	-1.21e+00	1.00e+00	4.23e-02
PTR	-1.31e-07	9.99e-05	-1.31e-03	1.00e+00	-3.54e+01	3.86e+01	-9.18e-01	1.00e+00	2.38e-02
SS	4.78e-05	9.87e-05	4.84e-01	1.00e+00	-4.76e+01	3.81e+01	-1.25e+00	1.00e+00	5.30e-02
SLF	6.46e-05	1.18e-04	5.49e-01	1.00e+00	-3.50e+01	4.54e+01	-7.71e-01	1.00e+00	2.80e-02

Table 3: General linear model results using age and inverse of temperature as co-variables to try identify influences on the fractional anisotropy values. Significant association is indicated in bold

Variable	Coef. (age)	S.E. (age)	t-value (age)	$p_{corr.}$ (age)	$\text{Coef.}_{1/T}$	$\text{S.E.}_{1/T}$	t-value $_{1/T}$	$p_{corr.1/T}$	R^2
THAL	-3.587e-04	4.503e-04	-7.965e-01	1.000e+00	-1.295e+02	1.605e+02	-8.070e-01	1.000e+00	3.569e-02
**CAU**	**2.777e-03**	**8.191e-04**	**3.391e+00**	**3.259e-02**	3.981e+02	2.918e+02	1.364e+00	1.000e+00	2.861e-01
BRST	2.068e-04	4.512e-04	4.584e-01	1.000e+00	-2.436e+01	1.608e+02	-1.515e-01	1.000e+00	8.110e-03
HIP	1.215e-04	3.697e-04	3.286e-01	1.000e+00	3.776e+00	1.317e+02	2.867e-02	1.000e+00	3.476e-03
AMY	6.331e-04	2.509e-04	2.523e+00	2.377e-01	2.409e+02	8.941e+01	2.695e+00	1.916e-01	2.819e-01
ACC	1.435e-03	7.807e-04	1.837e+00	8.332e-01	-9.680e+01	2.782e+02	-3.480e-01	1.000e+00	1.068e-01
ICP	-2.786e-04	5.580e-04	-4.993e-01	1.000e+00	-1.915e+02	1.988e+02	-9.629e-01	1.000e+00	3.355e-02
PPLIC	1.480e-03	4.666e-04	3.172e+00	5.448e-02	2.906e+02	1.663e+02	1.748e+00	1.000e+00	2.783e-01
RPIC	-2.750e-04	5.372e-04	-5.119e-01	1.000e+00	6.305e+01	1.914e+02	3.294e-01	1.000e+00	1.328e-02
ACR	-3.614e-04	4.423e-04	-8.171e-01	1.000e+00	-9.744e+00	1.576e+02	-6.182e-02	1.000e+00	2.112e-02
PTR	-1.502e-03	6.262e-04	-2.398e+00	2.948e-01	-1.275e+01	2.231e+02	-5.714e-02	1.000e+00	1.578e-01
SS	-1.075e-03	5.585e-04	-1.924e+00	7.624e-01	2.042e+01	1.990e+02	1.026e-01	1.000e+00	1.096e-01
SLF	-3.356e-04	5.044e-04	-6.653e-01	1.000e+00	-1.640e+02	1.797e+02	-9.125e-01	1.000e+00	3.564e-02

Table 4: Mean values for QSM, paramagnetic, diamagnetic, and a values in all ROIs analyzed

	χ **(ppm)**		χ_{para} **(ppm)**		χ_{dia} **(ppm)**		**a (Hz/ppm)**	
**ROI**	**mean**	**std**	**mean**	**std**	**mean**	**std**	**mean**	**std**
THAL	0.004	0.014	0.036	0.013	-0.028	0.008	575.19	57.84
CAU	0.045	0.018	0.058	0.021	-0.02	0.008	651.60	70.72
PUT	0.06	0.028	0.085	0.03	-0.025	0.006	667.04	54.17
PAL	0.083	0.037	0.1	0.039	-0.03	0.011	656.86	76.62
BRST	-0.004	0.01	0.027	0.009	-0.026	0.005	569.76	44.33
HIP	0.012	0.015	0.033	0.014	-0.022	0.006	527.99	59.44
AMY	0.002	0.013	0.027	0.011	-0.024	0.009	533.72	77.70
ACC	0.015	0.022	0.037	0.015	-0.022	0.009	609.96	77.44
SN	0.087	0.037	0.096	0.028	-0.022	0.008	642.98	56.98
RN	0.095	0.037	0.097	0.032	-0.021	0.008	662.40	68.13
ICP	-0.001	0.011	0.025	0.01	-0.021	0.008	626.01	70.01
PLIC	-0.027	0.013	0.03	0.02	-0.053	0.01	554.06	89.30
RPIC	-0.009	0.013	0.025	0.011	-0.033	0.006	686.81	38.48
ACR	-0.004	0.01	0.017	0.008	-0.023	0.006	709.41	35.15
PTR	-0.017	0.012	0.018	0.008	-0.035	0.01	649.61	59.00
SS	-0.012	0.012	0.022	0.008	-0.031	0.006	694.18	34.93
SLF	-0.001	0.01	0.024	0.009	-0.022	0.006	706.64	40.86

Table 5: Standard deviation, slope, and intercept for the linear regression of susceptibility values and age

	χ			χ_{para}			χ_{dia}		
	**std**	**slope**	**intercept**	**std**	**slope**	**intercept**	**std**	**slope**	**intercept**
**THAL**	1.36E-04	2.63E-06	4.36E-03	1.33E-04	-5.55E-05	3.98E-02	7.82E-05	-6.87E-07	-2.77E-02
**CAU**	1.82E-04	1.31E-04	3.62E-02	2.09E-04	3.16E-04	3.80E-02	6.59E-05	-2.91E-04	-1.04E-03
**PUT**	2.53E-04	8.51E-04	4.06E-03	2.55E-04	9.75E-04	2.12E-02	4.76E-05	-2.19E-04	-1.08E-02
**PAL**	3.78E-04	2.24E-05	8.29E-02	3.89E-04	-6.91E-07	1.01E-01	8.55E-05	-4.34E-04	-7.60E-04
**BRST**	1.05E-04	-3.08E-06	-3.67E-03	9.62E-05	-7.98E-06	2.75E-02	5.45E-05	-3.98E-05	-2.36E-02
**HIP**	1.52E-04	1.88E-04	-2.84E-04	1.36E-04	1.34E-04	2.47E-02	6.36E-05	9.52E-07	-2.15E-02
**AMY**	1.30E-04	3.01E-05	6.06E-05	1.05E-04	2.26E-04	1.25E-02	9.34E-05	-4.05E-05	-2.13E-02
**ACC**	2.21E-04	-6.58E-05	1.94E-02	1.55E-04	6.81E-05	3.29E-02	8.52E-05	-3.04E-04	-1.62E-03
**SN**	3.71E-04	-5.68E-04	1.26E-01	2.71E-04	-5.49E-04	1.34E-01	6.97E-05	-2.16E-04	-7.43E-03
**RN**	3.76E-04	3.63E-04	7.12E-02	3.21E-04	6.24E-05	9.38E-02	7.37E-05	-8.41E-05	-1.49E-02
**ICP**	1.07E-04	1.23E-04	-9.39E-03	1.02E-04	8.04E-05	1.97E-02	8.09E-05	1.17E-04	-2.93E-02
**PLIC**	1.33E-04	7.28E-05	-3.12E-02	1.96E-04	3.44E-04	7.53E-03	1.00E-04	-2.05E-04	-3.93E-02
**RPIC**	1.34E-04	-3.34E-05	-6.14E-03	1.06E-04	4.80E-05	2.22E-02	6.15E-05	-1.17E-04	-2.46E-02
**ACR**	1.04E-04	8.27E-05	-9.33E-03	8.19E-05	3.00E-05	1.54E-02	5.44E-05	1.08E-04	-3.03E-02
**PTR**	1.21E-04	1.40E-04	-2.59E-02	7.98E-05	6.47E-06	1.77E-02	9.24E-05	1.79E-04	-4.64E-02
**SS**	1.24E-04	7.25E-05	-1.64E-02	8.19E-05	6.24E-05	1.76E-02	6.40E-05	2.68E-05	-3.24E-02
**SLF**	9.86E-05	7.47E-05	-6.09E-03	9.65E-05	6.36E-05	2.03E-02	5.81E-05	2.31E-05	-2.33E-02

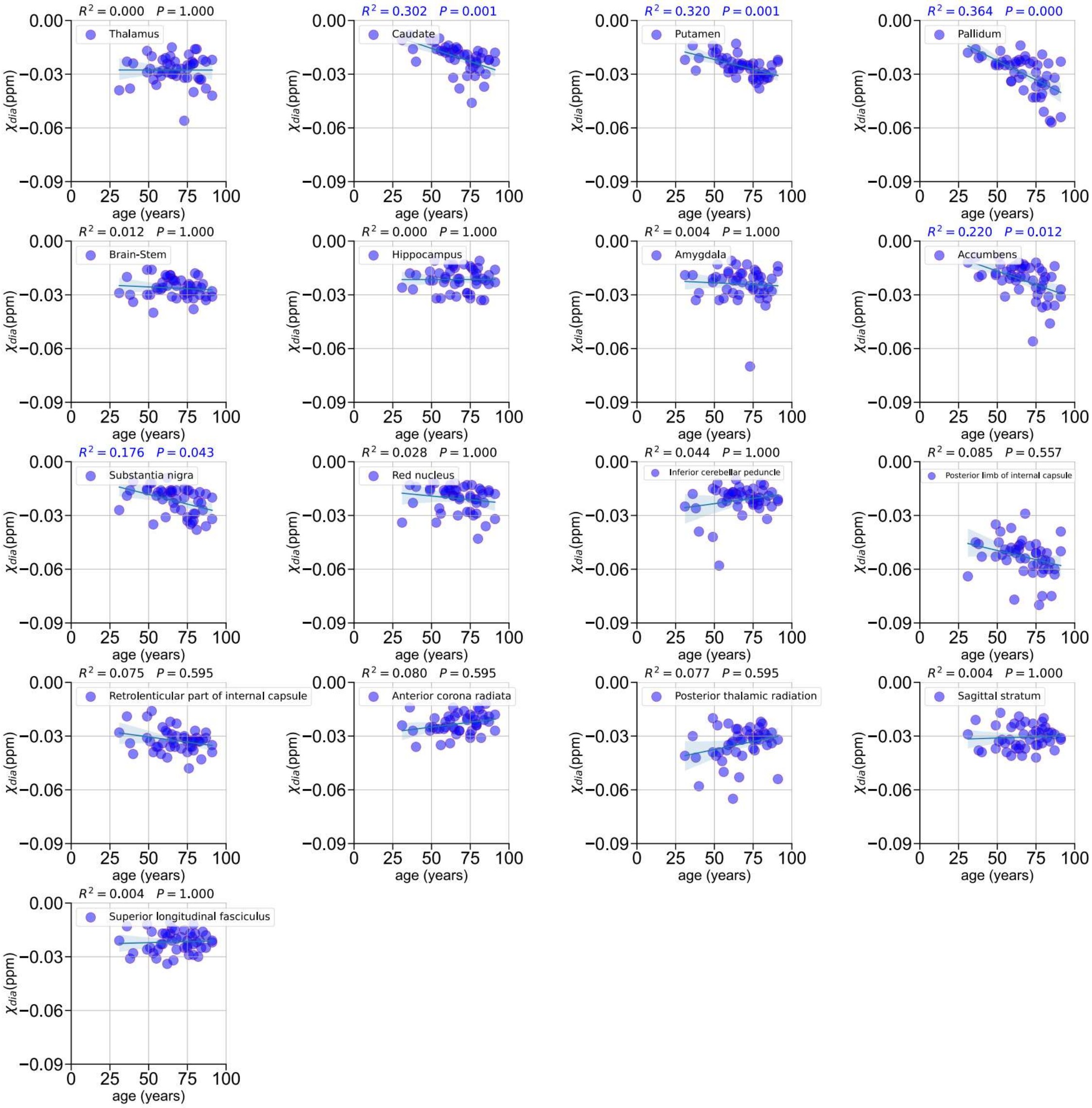

Figure 8: Diamagnetic susceptibility values vs age for different ROIs

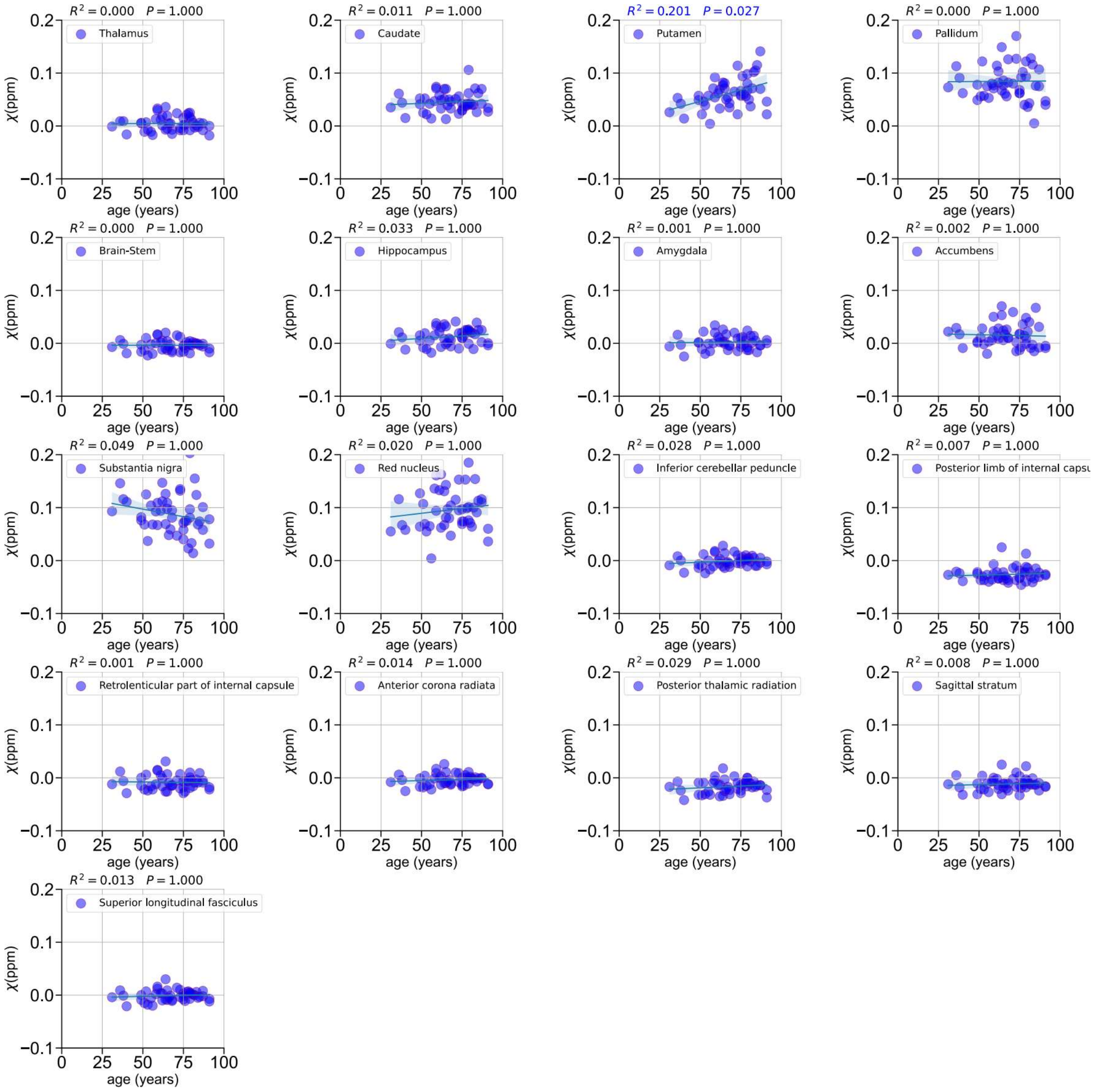

Figure 9: QSM susceptibility values vs age for different ROIs

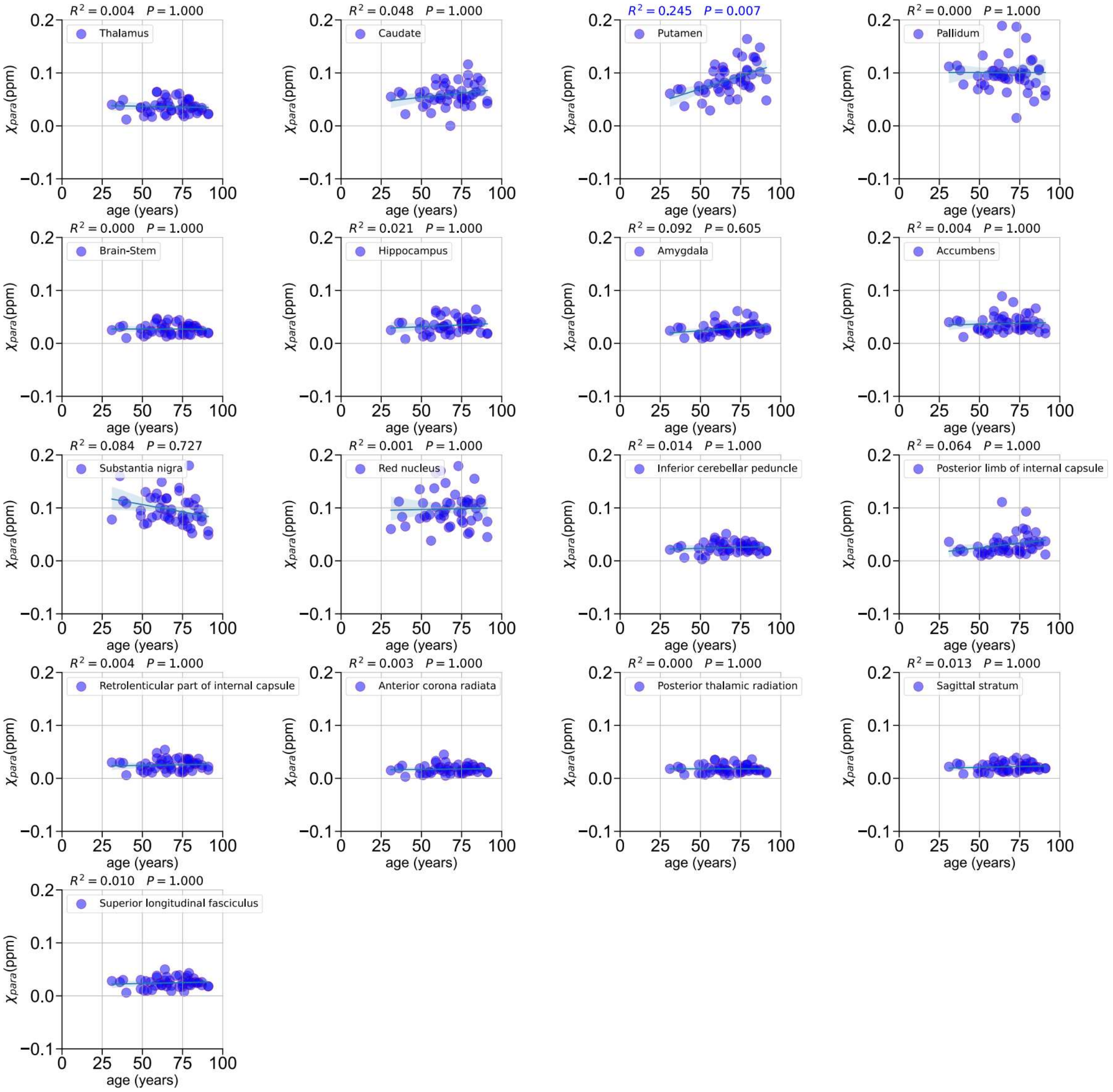

Figure 10: Paramagnetic susceptibility values vs age for different ROIs

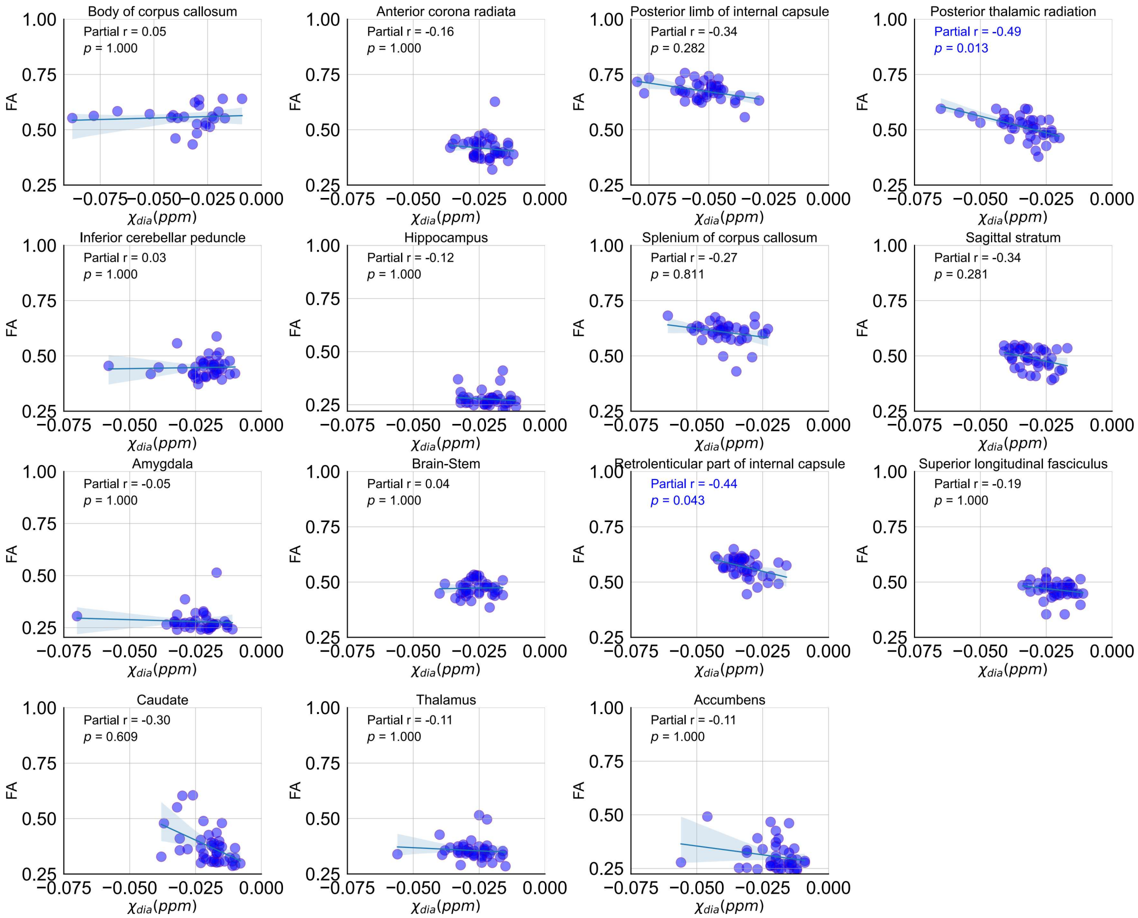

Figure 11: Diamagnetic susceptibility values vs FA for different ROIs, using age as covariate

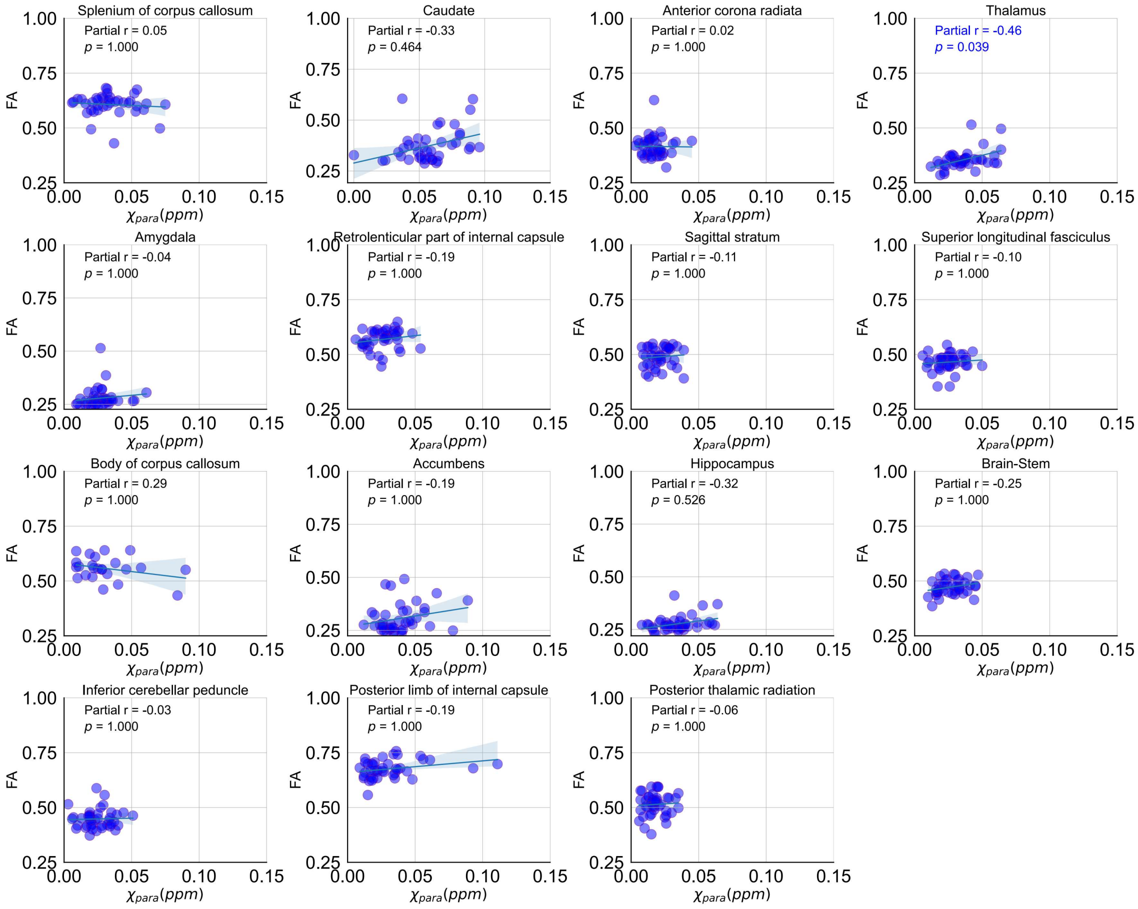

Figure 12: Paramagnetic susceptibility vs FA for different ROIs

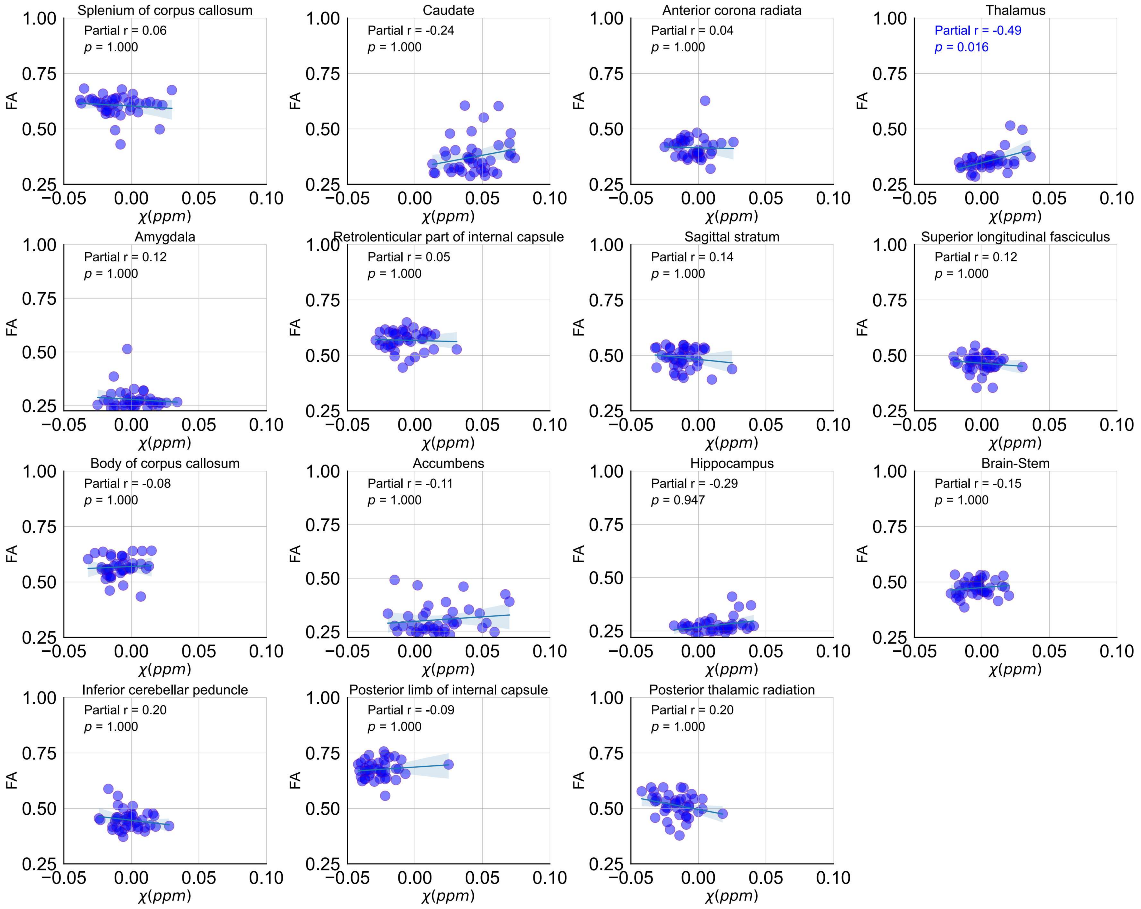

Figure 13: QSM susceptibility vs FA for different ROIs
